# Abnormally low prolactin levels in schizophrenia patients after switching to aripiprazole in a randomized trial: a biomarker for rebound in psychotic symptoms?

**DOI:** 10.1186/s12888-020-02957-7

**Published:** 2020-11-23

**Authors:** Ya-Wen Jen, Tzung-Jeng Hwang, Hung-Yu Chan, Ming H. Hsieh, Chen-Chung Liu, Chih-Min Liu, Hai-Gwo Hwu, Ching-Hua Kuo, Yi-Ting Lin, Yi-Ling Chien, Wei J. Chen

**Affiliations:** 1 Institute of Epidemiology and Preventive Medicine, College of Public Health, National Taiwan University, Taipei, Taiwan; 2 Department of Psychiatry, College of Medicine and National Taiwan University Hospital, National Taiwan University, Taipei, Taiwan; 3 Office of Superintendent, Taoyuan Psychiatric Center, Ministry of Health and Welfare, Taoyuan City, Taiwan; 4 School of Pharmacy, College of Medicine, National Taiwan University, Taipei, Taiwan; 5 Centers for Genomic and Precision Medicine, National Taiwan University, Taipei, Taiwan; 6 Center for Neuropsychiatric Research, National Health Research Institutes, 17 Xu-Zhou Road, Taipei, 100 Miaoli Taiwan

**Keywords:** Aripiprazole, Prolactin, Psychotic rebound, Biomarker, Schizophrenia

## Abstract

**Background:**

Switching to aripiprazole from other antipsychotics can avoid antipsychotic-induced hyperprolactinemia but may result in an abnormally low prolactin level. This study aimed to assess whether the aripiprazole-induced abnormally low prolactin level was a biomarker for subsequent rebound of positive symptoms in schizophrenia patients.

**Methods:**

Participants were 63 patients in an 8-week trial of switching to aripiprazole, in which preswitching antipsychotics were maintained for the first 2 weeks and aripiprazole was fixed at 15 mg orally throughout the trial. A prolactin level of < 3.7 ng/ml was defined as abnormally low, and an increase of two or more points in the positive subscore of the Positive and Negative Syndrome Scale at two adjacent ratings was defined as a psychotic rebound.

**Results:**

Among 63 patients, 25 (39.7%) had an abnormally low prolactin level and 21 (33.3%) had a psychotic rebound after switching to aripiprazole. In patients with abnormally low prolactin levels, 48.0% of them had a rebound in psychotic symptoms, whereas in those without abnormally low prolactin levels 23.7% did so. Multivariable logistic regression analysis with adjustment for sex, early age at onset, and preswitching medications revealed that abnormally low prolactin levels were associated with psychotic rebound (adjusted odds ratio = 3.55, 95% confidence interval = 1.02, 12.5). Furthermore, there was concurrency between the trend of the cumulative proportion of patients having an abnormally low prolactin level and that of the cumulative proportion of patients having a rebound in psychotic symptoms.

**Conclusions:**

An abnormally low prolactin level after switching to aripiprazole in schizophrenia patients was a potential warning sign of a psychotic rebound. Hence, monitoring of prolactin levels after switching to aripiprazole may help avoid such rebound in schizophrenia.

**Trial registration:**

NCT00545467; Date of registration: 17/10/2007.

**Supplementary Information:**

**Supplementary information** accompanies this paper at 10.1186/s12888-020-02957-7.

## Background

Aripiprazole, an atypical antipsychotic with unique pharmacological properties of dopamine D2 and serotonin 1A partial agonism and serotonin 2A antagonism [1], can ameliorate schizophrenia symptoms with fewer adverse effects, e.g., a low potential for weight gain and extrapyramidal symptoms [2], and can reverse antipsychotic-associated hyperprolactinemia [3–7]. There is an ongoing debate as to whether switching patients to aripiprazole from other antipsychotics would exacerbate psychosis, a topic first covered in a systematic review of case reports [8] and in a meta-analysis of randomized trials [9]. Some trials comparing the efficacy of aripiprazole with other antipsychotics in Asian populations have reported worsening psychosis as an adverse event [10, 11].

The partial agonism of aripiprazole on the dopaminergic neurons in the mesolimbic system might be too intense for those patients who had increased postsynaptic D2 receptor density in response to previous antipsychotics’ blocking [12, 13]. This may lead not only to a worsening in psychotic symptoms but also to a lowering of prolactin level (PRL) through inhibition by dopamine in the tuberoinfundibular pathway [14]. A review of 11 trials of aripiprazole in children and adolescents found, using 2 ng/mL as the threshold, that approximately 60% of participants exhibited subnormal PRL [15]. Case reports and retrospective studies among adult schizophrenia patients receiving aripiprazole also showed abnormally low PRL, with varying thresholds [16–19].

Intriguingly, the PRL of schizophrenia outpatients became lower right before relapse compared to those during the stable period [20, 21]. Unmedicated first-episode patients had a low-to-normal level of prolactin, particularly for a subtype of patients with positive symptoms [22–24]. The association of lower PRL with positive symptoms was in line with the postulation that dopamine transmission was increased within this particular subgroup of schizophrenia patients [25, 26]. Such an association might serve as a potential biomarker for antipsychotic-induced dopamine supersensitivity [13]. However, none of the existing studies have investigated longitudinally the relationship between abnormally low PRL and a quantitative increase in psychotic symptoms after switching to aripiprazole.

To fill in the gap in the literature, we turned to a trial of schizophrenia patients with repeated measurements for PRL and positive symptoms after switching to aripiprazole. The aims of the study were (1) to investigate the prevalence of abnormally low PRL after switching to aripiprazole in patients with schizophrenia, (2) to evaluate whether such an abnormally low PRL was associated with rebound in psychotic symptoms, and (3) to evaluate the concurrency of both the trend of cumulated proportion of abnormally low PRL and the trend of cumulated proportion of rebound in psychotic symptoms during the trial.

## Methods

### Participants

Study subjects were patients with schizophrenia (*n* = 63) in a previous clinical trial assessing fast versus slow strategies for switching to aripiprazole from other antipsychotics conducted at National Taiwan University Hospital, Taoyuan Psychiatric Center, and Ju-Shan Hospital from September 2007 to July 2009 [27]. More details about the trial have been reported [27]. Briefly, during this 8-week open-label trial with a randomization ratio of 1:1, there were 2 weeks when both preswitching antipsychotics and aripiprazole were administered simultaneously, followed by either fast (tapering within 1 week) or slow (tapering within 4 weeks) switching to aripiprazole alone. The dose of preswitching antipsychotics was maintained for the first 2 weeks, and the full dose of aripiprazole, 15 mg orally, was fixed throughout the study.

Men and nonpregnant, nonlactating women were eligible for enrollment in the study if they were aged 18 to 65 years with a primary diagnosis of schizophrenia or schizoaffective disorder according to the Diagnostic and Statistical Manual of Mental Disorders, Fourth Edition. The patients were also required to be chronic and stable in terms of their disease, as defined by taking a stabilized dose of a typical single oral antipsychotic or atypical antipsychotic for at least 1 month before study entry. The preswitching antipsychotics were classified by their risk of prolactin elevation into high (risperidone and amisulpride), medium (first generation antipsychotics and zotepine), and low risk (Olanzapine, Ziprasidone, and Quetiapin), and their doses were expressed in terms of chropromazine equivalences [28].

Permuted block randomization (generated by computer) with a block size of 4 was performed with stratification by hospital to balance the distribution of the 2 groups in each hospital. Consecutively numbered, sealed, identical opaque envelopes with a randomization code inside according to the random number list was prepared by the central office. The envelope and relevant questionnaires and rating scales for each subject was placed in a folder. These folders were then sent to the three study sites where the clinical staff performed the study according to the order of the sealed envelopes. By concealing the allocation, selection bias could be avoided. Because the psychiatrists who recruited patients would explain the regimen, provide suggestions in answering patients’ phone calls about side effects, and conduct efficacy assessments, an open-label rather than double blinded trial was adopted for this study.

Since the participants were in chronic and stable condition, all patients provided informed written consent after the study procedure had been fully explained to them, and the written consent from Legally Authorized Representatives of the patients was pursued only if a patient was judged to have difficulty in understanding the study. The study was approved by the institutional review board of the participating hospitals, including the Research Ethics Committee of the National Taiwan University Hospital (NTUH-REC no. 200705030 M) and the Ethical Review Committee of Taoyuan Mental Hospital, Department of Health, Executive Yaun (C200705021), and has therefore been performed in accordance with the ethical standards laid down in the 1964 Declaration of Helsinki. This study adheres to the CONSORT guidelines for reporting results from clinical trials, and it has been registered with ClinicalTrials.gov (number NCT00545467; Date of First Posted:17/10/2007).

The Chinese version of the Positive and Negative Syndrome Scale (PANSS) [29] was used to assess treatment efficacy in this trial. The interrater reliability for the PANSS items was acceptable to good, with intraclass correlations ranging from 0.50 to 0.86 [27]. For each patient, the treatment efficacy was assessed at 5 time points, i.e., baseline, the 7th Day, the 14th Day, the 28th Day, and the 56th Day, by the same psychiatrist throughout the trial. The primary efficacy parameter was the change in PANSS total score from baseline, whereas the secondary efficacy parameters included changes in 3 PANSS subscores (positive, negative, general psychopathology).

### Defining abnormally low PRL

Patients’ venous blood was drawn in the morning under fasting conditions before drug administration at 3 time points (baseline, the 14th Day, and the 56th Day) during the trial. The serum PRL was measured using commercially available kits (“RADIM” Prolactin MAIAclone, Pomezia (Roma), Italy), which had a detection range of 0.3–500 ng/ml.

Based on the normal range of PRL (3.7–16.3 ng/ml) reported in an Asian population [19], we used a PRL of < 3.7 ng/ml to define abnormally low PRL at either the 14th Day or 56th Day. Hence, patients needed to have at least one follow-up measurement of prolactin level to be included in this study. Out of 79 participants enrolled in the trial, 63 patients were eligible for this study. Detailed information about the changes in PRL as well as PANSS positive subscores along the time axis of days for each of 63 participants is shown in Supplementary Figure S1.

For sensitivity analysis, we reran the analysis by applying different criteria for an abnormally low PRL used in another study in an Asian population: < 3.57 ng/mL for males and < 6.12 ng/mL for females [18].

### Defining rebound in psychotic symptoms

A rebound in psychotic symptoms was based on an increase in a PANSS positive subscore derived from the five-factor model of PANSS, i.e., delusions (p1), hallucinations (p3), grandiosity (p5), suspiciousness (p6), and unusual thought content (g9), with each item rated from 1 (absent) to 7 (severe) and having an intraclass correlation coefficient ranging from 0.79 to 0.92 [30, 31]. Hence, the 5-item positive subscore ranged from 5 to 35. Because aripiprazole requires up to 10 to 14 days of treatment to reach a steady state [32], the PANSS rating at the 7th Day was not used in our evaluation of rebound in psychotic symptoms. Although many previous trials used an increase of 20% in PANSS total score to define a psychotic exacerbation [33], the relativity of the criterion rendered it difficult to apply or interpret in a trial with multiple measurements. Since the mean positive subscore of this sample was 10.3, we decided to adopt an absolute criterion by using 20% of the mean, i.e., 2 points, to define a rebound in psychotic symptoms as an increase of two or more points in the PANSS positive subscore at two adjacent ratings, i.e., from baseline to the 14th Day, from the 14th Day to the 28th Day, or from the 28th Day to the 56th Day.

For the sensitivity analysis, we conducted the analysis using three different criteria in assessing the rebound in psychotic symptoms: 1) an increase of one or more points; 2) an increase of three or more points; and 3) an increase of 20% or more points in the PANSS positive subscore.

### Statistical analysis

Group comparisons were performed using the Mann-Whitney test for continuous variables and Fisher’s exact test for categorical variables. Multivariable logistic regression analysis of the rebound in psychotic symptoms in the presence of an abnormally low PRL was conducted with adjustment for sex, early age at onset, and preswitching medication, with adjusted odds ratios (aORs) and their 95% confidence intervals (95% CI) presented. To evaluate the concurrency between the trend of the cumulated proportion of having an abnormally low PRL and the trend of the cumulated proportion of having a rebound in psychotic symptoms during the trial, we first estimated the linear coefficient for each trend in the whole sample and then compared the two coefficient estimates. All analyses were performed using SAS software version 9.4 (SAS Institute Inc., NC, USA) and R-software version 3.4.0 (R Development Core Team, 2016). A two-sided *P* value of less than 0.05 was considered statistically significant.

## Results

### Abnormally low PRL

**Fig. 1:**
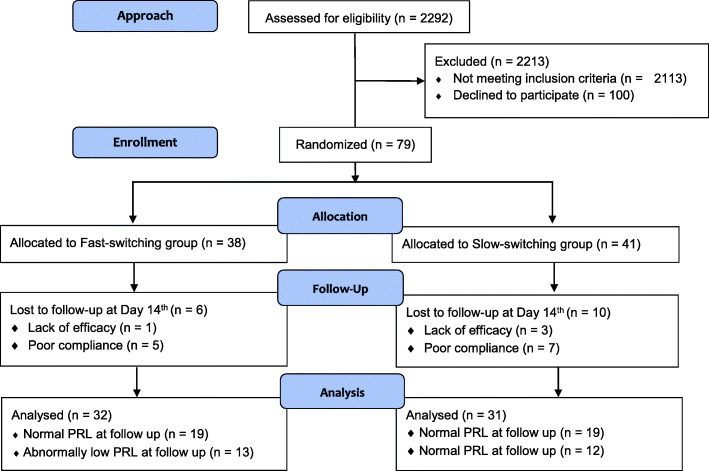
CONSORT flow diagram for the study

Figure 1 shows the CONSORT flow diagram of the number of participants at each stage of the trial. Participants who had completed the Day 14th follow-up were included in the analyses. Of the 63 patients, 25 (39.7%) had an abnormally low PRL at least once after switching to aripiprazole. Compared with those with normal PRL, patients with an abnormally low PRL had a greater proportion of male sex, higher height, weight, and lower PRL throughout the trial (Table 1). The distribution of preswitching antipsychotics with disparate risk for prolactin elevation between patients with and without experiencing abnormally low PRL was similar. More detailed information on preswitching antipsychotics is shown in Supplementary Table S1. Otherwise, both groups had similar distributions in the remaining demographic and baseline clinical characteristics.

**Table 1:** Demographic and baseline clinical characteristics of participants in the trial of switching to aripiprazole by the grouping of prolactin levels at follow up

Characteristic	Total (*N* = 63)	Normal prolactin level at follow up (*N* = 38)	Abnormally low prolactin level at follow up (*N* = 25)	P^a^
Male, n (%)	26 (41.27)	10 (26.32)	16 (64.00)	0.003**
Age, years, (SD)	38.7 (11.29)	37.7 (12.25)	40.2 (9.69)	0.38
Body height, cm, mean (SD)	162.3 (8.83)	159.5 (7.77)	166.6 (8.78)	0.003**
Body weight, kg, mean (SD)	66.6 (11.96)	63.5 (12.53)	71.4 (9.36)	0.008**
Age of onset, years, mean (SD)^b^	27.7 (8.81)	28.8 (9.73)	26.1 (7.14)	0.44
Early age of onset, n (%) ^b^	8 (12.70)	6 (15.79)	2 (8.00)	0.46
Late dropout, n (%)	10 (15.87)	7 (18.42)	3 (12.00)	0.23
Prolactin level, ng/dL, mean (SD)				
Baseline	53.9 (61.38)	74.0 (69.94)	20.8 (20.35)	0.001**
14th Day	23.5 (35.15)	31.7 (38.81)	11.0 (24.51)	< 0.001**
56th Day^c^	6.9 (6.03)	10.0 (6.05)	2.4 (1.18)	< 0.001**
Positive subscore in PANSS at baseline, mean (SD)^d^	10.3 (4.48)	10.7 (4.54)	9.8 (4.43)	0.45
Switching strategy in previous trial, n (%)				0.88
Fast strategy	32 (50.79)	19 (50.00)	13 (52.00)	
Slow strategy	31 (49.21)	19 (50.00)	12 (48.00)	
Preswitching antipsychotics^e^				0.32
High risk of prolactin elevation (RIS or AMI), n (%)	16 (25.39)	12 (31.00)	4 (16.00)	
Medium risk of prolactin elevation (FGA or ZOT), n (%)	18 (28.57)	9 (24.00)	9 (36.00)	
Low risk (OLA or ZIP or QUE), n (%)	29 (46.03)	17 (45.00)	12 (48.00)	
Chlorpromazine equivalent dose, mean (SD)	289.0 (231.80)	289.1 (260.80)	288.9 (184.30)	0.71

*Abbreviation*: *RSI* Risperidone, *AMI* Amisulpride, *FGA* First generation antipsychotics, *ZOT* Zotepine, *OLA* Olanzapine, *ZIP* Ziprasidone, *QUE* Quetiapine

^a^Fisher’s exact test (for categorical variables) or Mann-Whitney test (for continuous variables) in comparing the 2 groups. ** *p* < .01

^b^Data missing for 1 patient

^c^Data missing for 10 patients; i.e., including only patients completing the trial (*n* = 52)

^d^Including delusions (p1), hallucinations (p3), grandiosity (p5), suspiciousness (p6), and unusual thought content (g9)

^e^Following the classification by Buchanan et al. (2010) [28]

### Rebound in psychotic symptoms

During the follow-up, 21 (33.3%) showed at least one rebound in psychotic symptoms. At baseline, patients with a psychotic rebound had higher scores in the PANSS positive subscore and a greater likelihood of receiving first-generation antipsychotics compared to those with no rebound in positive symptoms (Table 2). Otherwise, there were no significant differences between the two groups in the remaining demographic and clinical characteristics.

**Table 2:** Comparison of demographic and baseline clinical characteristics of patients with schizophrenia in the trial of switching to aripiprazole between rebound and no rebound in positive subscores (*N* = 63)

Characteristic	Rebound in positive subscores (*N* = 21)	No rebound in positive subscores (*N* = 42)	P^a^
Male, n (%)	9 (42.86)	17 (40.48)	0.86
Age, years, (SD)	41.8 (10.60)	37.1 (11.44)	0.14
Body height, cm, mean (SD)	161.5 (7.19)	162.7 (9.61)	0.70
Body weight, kg, mean (SD)	68.4 (11.35)	65.8 (12.29)	0.52
Age of onset, years, mean (SD)^b^	26.6 (9.04)	28.2 (8.75)	0.30
Early age of onset, n (%) ^b^	2 (9.52)	6 (14.29)	0.71
Late dropout, n (%)	4 (19.05)	6 (14.29)	0.25
Prolactin level at baseline, ng/dL, mean (SD)	47.6 (69.48)	55.6 (57.63)	0.15
Positive subscore in PANSS at baseline, mean (SD)^c^	12.2 (4.41)	9.4 (4.26)	0.01*
Switching strategy in a previous trial, n (%)			0.72
Fast strategy	10 (47.62)	22 (52.38)	
Slow strategy	11 (52.38)	20 (47.62)	
Preswitching medication			0.04*
First generation antipsychotics, n (%)	12 (57.14)	13 (30.95)	
Second generation antipsychotics, n (%)	9 (42.86)	29 (69.05)	
Chlorpromazine equivalent dose, mean (SD)	321.4 (198.20)	272.8 (247.60)	0.27

*p < .05

^a^Fisher’s exact test (for categorical variables) or Mann-Whitney test (for continuous variables) in comparing the 2 groups

^b^Data missing for 1 patient

^c^Including delusions (p1), hallucinations (p3), grandiosity (p5), suspiciousness (p6), and unusual thought content (g9)

### Abnormally low PRL and rebound in psychotic symptoms

Patients with an abnormally low PRL had a greater likelihood of experiencing a rebound in psychotic symptoms than those without an abnormally low PRL (48.0% vs. 23.7%, *p* = 0.04). Table 3 displays the results of multivariable logistic regression analysis of the rebound in psychotic symptoms on patients’ abnormally low PRL with two different kinds of adjustments. For model 1, after adjustment for sex, early age at onset, and preswitching medication, having an abnormally low PRL at follow-up was significantly associated with a rebound in psychotic symptoms, with an aOR of 3.55 (95% CI: 1.02–12.5). For model 2, with further adjustment for baseline prolactin levels and PANSS positive subscore, the association remained significant (aOR = 4.95, 95% CI: 1.08–22.7). In addition, the interaction term between abnormally low PRL and sex on the rebound of psychotic symptoms was not significant.

**Table 3:** Multivariable logistic regression analysis of rebound in positive subscores on the abnormally low prolactin levels among schizophrenia patients participating in the trial of switching to aripiprazole (*N* = 63)

Variables	Rebound in positive subscores (*n* = 21)	No rebound in positive subscores (*n* = 42)	Multivariate-adjusted OR (95% CI)	
			Model 1	Model 2
Male, n (%), (ref. female)	9 (42.90)	17 (40.50)	0.57 (0.16–2.06)	0.42 (0.09–2.01)
Early age of onset, n (%), (ref. late age of onset)a	2 (9.52)	6 (14.29)	0.62 (0.10–3.94)	0.41 (0.06–2.82)
Preswitching medication				
First generation antipsychotics, n (%)	12 (57.10)	13 (31.00)	1.00 (reference)	1.00 (reference)
Second generation antipsychotics, n (%)	9 (42.90)	29 (69.00)	0.31 (0.10–0.98)	0.22 (0.06–0.82)
Prolactin level at baseline, ng/dL, mean (SD)	47.6 (69.48)	55.6 (57.63)	ˍ	1.00 (0.99–1.01)
Positive subscore in PANSS at baseline, mean (SD)b	12.2 (4.41)	9.4 (4.26)	ˍ	1.25* (1.07–1.46)
Abnormally low prolactin levels at follow up, n (%)	12 (57.10)	13 (31.00)	3.55* (1.02–12.5)	4.95* (1.08–22.7)
Interaction term, abnormally low prolactin levels × sex			0.66 (0.05–9.23)	0.38 (0.02–7.24)

Model 1: adjustment for sex, early age of onset, and preswitching medication

Model 2: Model 1 plus an adjustment for prolactin level and positive subscore in PANSS at baseline

*p < .05

^a^ ≤ 18 years old defined as early age of onset; One observation was deleted due to a missing value

^b^Including delusions (p1), hallucinations (p3), grandiosity (p5), suspiciousness (p6), and unusual thought content (g9)

Among patients with late drop out after 2 weeks of comedication, three out of three were due to a rebound in psychotic symptoms for the group with an abnormally low PRL, whereas the corresponding figure was one out of seven for the group without an abnormally low PRL.

### Trend of abnormally low PRL vs. trend of rebound in psychotic symptoms

**Fig. 2:**
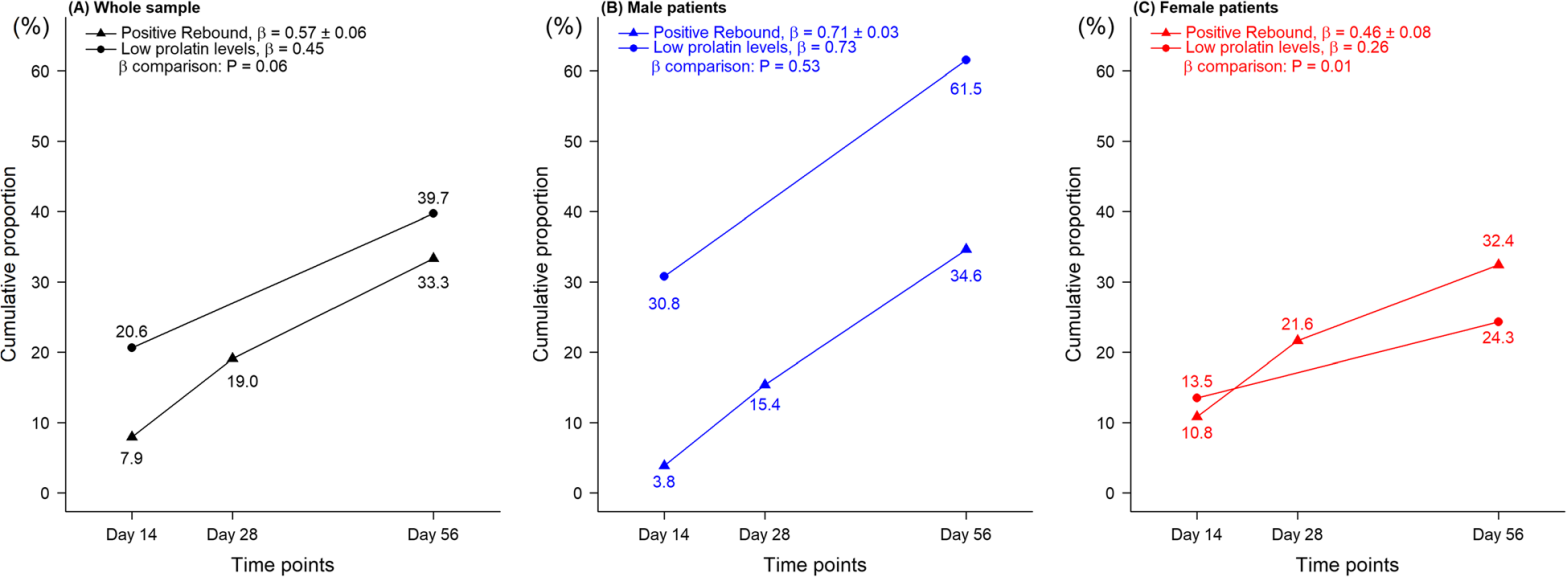
The cumulative proportion of patients with rebound in PANSS positive symptoms (▲) at follow-up measured time points, which are the 14th Day, 28th Day, and 56th Day, and of patients with abnormally low prolactin levels (●) at follow-up measured time points, which are the 14th Day and 56th Day. Concurrency of the two trends over time in the cumulative proportions is shown in **a** whole samples, *N* = 63, **b** male patients, *N* = 26, and **c** female patients, *N* = 37. The β coefficient presents the slope of the cumulated line. *P* value is from testing whether the β coefficient of positive rebound is different from that of low prolactin levels

The time trends of the cumulative proportions of having an abnormally low PRL among patients as well as that of having a rebound in psychotic symptoms are displayed in Fig. 2. For the whole sample (Fig. 2a), the two slopes were similar (β = 0.45 vs. 0.57, *p* = 0.06). When stratified by sex, the slope of having an abnormally low PRL was higher in males than in females (0.73 in Fig. 2b vs. 0.26 in Fig. 2c). However, there remained an increasing trend of having a rebound in psychotic symptoms (0.71 for males and 0.46 for females).

### Sensitivity analysis of different definitions

Regarding the criterion of abnormally low PRL, the results of using separate criteria for males (< 3.57 ng/mL) and females (< 6.12 ng/mL) are presented in Supplementary Table S2. The association between abnormally low PRL and rebound in psychotic symptoms failed to reach statistical significance, though the direction of the effect size estimates (1.3 for model 1 and 1.54 for model 2) was consistent with the original.

Regarding the criterion of a rebound in psychotic symptoms, we conducted the sensitivity analysis examining three other definitions (Supplementary Table S3-S5). Our results indicated that an abnormally low PRL would be associated with a rebound in psychotic symptoms that was defined using the less stringent criterion (aOR = 4.42, 95% CI: 1.29–15.2) and a relative criterion (aOR = 5.27, 95% CI: 1.33–20.9), but did not reach statistical significance with the rebound defined using the more stringent criterion (aOR = 2.89, 95% CI: 0.71–11.8), possibly due to the small number of patients meeting the criterion of abnormally low PRL.

## Discussion

This study aimed to evaluate the relationship between an abnormally low PRL and a rebound in psychotic symptoms after switching to aripiprazole in patients with schizophrenia. We found that an abnormally low PRL after switching to aripiprazole was associated with a rebound in psychotic symptoms, which was further supported in a multivariable logistic regression analysis with adjustments for sex, early age at onset, and preswitching medications. Furthermore, there was concurrency between the trend of the cumulative proportion of patients having an abnormally low PRL and that of the cumulative proportion of patients having a rebound in psychotic symptoms. These findings help shed new light on the potential role of an aripiprazole-induced abnormally low PRL as a warning sign of a rebound in psychotic symptoms and may have utility in the clinical management of chronic schizophrenia patients when switching to aripiprazole.

### Definition of abnormally low prolactin level

As an exploratory analysis, this study chose the criterion of abnormally low PRL based on the lower boundary of the prolactin range in an Asian population cited in a case report [19]. Despite the slight differences in the definition, the estimated prevalence of having an abnormally low PRL in this study (40% at the 56th Day) were similar to the existing studies, e.g., 45% in patients who had received combination therapy with aripiprazole (≤ 3 ng/mL) [16] and 44% in patients during a long retrospective follow-up period after aripiprazole use (≤ 3.57 ng/mL for males and ≤ 6.12 ng/mL for females) [18]. Nevertheless, in our sensitivity analysis, we found that the sex-specific criteria of abnormally low PRL were probably too loose for females (i.e., too many female patients fulfilled this criterion) to diminish the underlying association with the rebound in psychotic symptoms.

Since a greater proportion of patients experiencing an abnormally low PRL were males who tended to have less antipsychotic-induced elevation in PRL than female patients [34], our multivariable logistic regression model included patient’s sex in the covariates. The PRL at baseline might result from differential prolactin-elevation potencies of preswitching antipsychotics, e.g., risperidone and amisulpride that have been reported to have a higher likelihood of hyperprolactinemia [28]. Nevertheless, the distributions of preswitching antipsychotics with disparate risk for prolactin elevation were not different between patients with and without experiencing abnormally low PRL.

### How to define rebound in psychotic symptoms

We selected PANSS items to define a psychotic rebound based on the Positive factor (P1, P3, P5, P6, and G9) in an empirically derived five-factor structure of the Chinese version of PANSS [31]. Previous trials commonly used an increase of 20% in the Brief Psychiatric Rating Scale total score or in PANSS total score throughout the trial to define quantitatively a psychotic exacerbation [33]. Others selected a specific threshold of certain scales, e.g., a Clinical Global Impressions scale-severity score ≥ 3, 4, or 6; or a Clinical Global Impressions scale-change score ≥ 3, 4, or 6 [33]. Some trials used part of the PANSS positive subscale (P1, P2, P3, P5, and P6) to define relapse, mainly based on clinical judgment [35, 36]. We did not use a proportional change to define a psychotic rebound is that we intended to develop a measurement that has immediate clinical implications during short intervals. Thus, clinicians can be alerted of a positive rebound if a patient was found to have an increase of two or more points in the PANSS positive subscore. Nevertheless, the direction and effect size estimates remain similar in our sensitivity analyses.

### Abnormally low prolactin and rebound in psychotic symptoms

An important finding of this study is that an abnormally low PRL after switching to aripiprazole might precede or accompany a psychotic rebound. Previous studies either focused on how to use aripiprazole to deal with antipsychotic-induced hyperprolactinemia [3–7], or whether aripiprazole might worsen psychosis in the long run [8, 9], with none evaluating the dynamic relationship between aripiprazole-induced abnormally low PRL and a rebound in psychotic symptoms.

There are some plausible explanations for this association. Long-term antipsychotic treatment will increase postsynaptic D2/D3 receptor density and hence induce supersensitivity of those receptors [12, 13]. Since all the participants in this study were chronic stable patients, they would be likely to develop some supersensitive dopamine receptors. Upon switching to aripiprazole, those patients with dopamine receptor supersensitivity would experience abnormally low PRL through the tuberoinfundibular pathway and a rebound in psychotic symptoms through the mesolimbic system. The concomitant action of dopamine on the two pathways has been demonstrated in the action of apomorphine, also a DRD2 agonist [37, 38]. Lower PRL has been related to more psychotic symptoms [17, 18, 23, 24, 39, 40]. Hence, an abrupt lowering of PRL to an abnormally low level could be considered as a biomarker or warning sign for relapse in psychosis.

Another possibility is that aripiprazole may disrupt the hypothalamus’ secretion of thyroid-releasing hormone, resulting in a decrease in plasma levels of prolactin-releasing hormone and thyroid-stimulating hormone [19]. Hence, a mere lowering in prolactin may not be indicative of the partial agonist effect of aripiprazole, which may explain why some patients with abnormally low PRL did not experience a rebound in psychotic symptoms. Antipsychotics might alter pituitary gland volumes through the hypothalamic-pituitary-adrenal (HPA) axis, with smaller pituitary volumes being associated with psychotic relapses [41]. Taken together, future investigation is warranted to measure the activities of the HPA axis after switching to aripiprazole to elucidate the brain mechanism involved in the association of abnormally low PRL after switching to aripiprazole with an increased risk of psychotic rebound.

Our results have a practical implication in clinical management for switching to aripiprazole, which is a common strategy to ameliorate the antipsychotic-induced hyperprolactinemia in clinical practice. A routine monitoring of PRL after switching to aripiprazole can help alert the clinician of the need for further adjustment to avoid a relapse of psychotic symptoms, e.g., decreasing the dosage of aripiprazole or adding other atypical antipsychotic when the PRL is approaching the abnormally low threshold.

This study has other limitations. First, this study was of an exploratory nature and had a relatively small sample size. Since PRLs may rise and fall for many other reasons, they would be more informative if the sample size is larger such that potential confounders could be adequately controlled for. Second, patients underwent combination therapy for the first 2 weeks, during which the PRL was still affected by the preswitching antipsychotics. Thus, using the PRL at the 14th Day could not predict a rebound in psychotic symptoms during the remaining follow-up period. Third, the inclusion of preswitching antipsychotics as a covariate in our logistic regression analysis might not adjust adequately for its confounding effect, since only the information on the immediate preswitching antipsychotics was used. Nevertheless, the misclassification in preswitching antipsychotics was likely nondifferential and hence resulted in a bias toward the null. Fourth, the sequence of abnormally low PRL and rebound in psychotic symptoms cannot be explicitly disentangled in this study since the PRL was measured only twice after baseline. Finally, aripiprazole’s role as agonist and antagonist depends on dose. Under 15 mg/day, it is usually psychotomimetic (agonist); over 15 mg/day, it usually acts as an antipsychotic (antagonist). The 15 mg dose used in this study could mean an enhanced psychotomimetic action. Research using a higher dose might be able to resolve this issue.

## Conclusions

In conclusion, our findings revealed that an abnormally low PRL after switching to aripiprazole in schizophrenia patients might be a potential warning sign of a rebound in psychotic symptoms. Therefore, routine monitoring of PRL after switching to aripiprazole may be useful in adjusting the treatment strategy to avoid a rebound in psychotic symptoms in chronic schizophrenia patients.

## Supplementary Information

**Additional file 1: Table S1.** Distribution of the pre-switching antipsychotics and the prolactin level at baseline between the two groups classified by the grouping of prolactin levels at follow up. **Table S2.** Using separate criterion on sex (< 3.57 ng/mL for men; < 6.12 ng/mL for women) to evaluated the correlation between the abnormal decline in prolactin levels and rebound in psychotic symptoms in patients with schizophrenia. **Table S3.** Using less stringent criterion of rebound in positive symptoms (PANSS positive subscores increased at least 1 points between adjacent time points) to evaluated the correlation between the abnormal decline in prolactin levels and rebound in psychotic symptoms in patients with schizophrenia. **Table S4.** Using more stringent criterion of rebound in psychotic positive symptoms (PANSS positive subscores increased at least 3 points between adjacent time points) to evaluated the correlation between the abnormal decline in prolactin levels and rebound in psychotic symptoms in patients with schizophrenia. **Table S5.** Multivariable logistic regression analysis of correlation between the abnormal decline in prolactin levels and rebound in psychotic positive symptoms (increasing at least 20% between adjacent time points) in patients with schizophrenia (*N* = 63). **Figure S1.** Each panel shows each participant’s changes in prolactin serum levels (baseline, 14th Day, and 56th Day) and PANSS positive subscores (baseline, 7th Day, 14th Day, 28th Day, and 56th Day) along the time axis of days in our group’s previous trial. Blue line presents the trend of prolactin serum levels, and red line presents the trend of PANSS positive subscore.

## Data Availability

Data and materials are available on request.

## Abbreviations

PRL: Prolactin level
PANSS: Positive and Negative Syndrome Scale
aORs: Adjusted odds ratios
CI: Confidence intervals
HPA: Hypothalamic-pituitary-adrenal

## References

[1] Jordan S, Koprivica V, Chen R, Tottori K, Kikuchi T, Altar CA (2002). The antipsychotic aripiprazole is a potent, partial agonist at the human 5-HT1A receptor. Eur J Pharmacol.

[2] Bhattacharjee J, El-Sayeh HG. Aripiprazole versus typical antipsychotic drugs for schizophrenia. Cochrane Database Syst Rev. 2008:CD006617. 10.1002/14651858.CD006617.pub3 PMC7052752 18646161

[3] Shim JC, Shin JG, Kelly DL, Jung DU, Seo YS, Liu KH (2007). Adjunctive treatment with a dopamine partial agonist, aripiprazole, for antipsychotic-induced hyperprolactinemia: a placebo-controlled trial. Am J Psychiatry.

[4] Li X, Tang Y, Wang C (2013). Adjunctive aripiprazole versus placebo for antipsychotic-induced hyperprolactinemia: meta-analysis of randomized controlled trials. PLoS One.

[5] Ziadi Trives M, Bonete Llacer JM, Garcia Escudero MA, Martinez Pastor CJ (2013). Effect of the addition of aripiprazole on hyperprolactinemia associated with risperidone long-acting injection. J Clin Psychopharmacol.

[6] Chen JX, Su YA, Bian QT, Wei LH, Zhang RZ, Liu YH (2015). Adjunctive aripiprazole in the treatment of risperidone-induced hyperprolactinemia: a randomized, double-blind, placebo-controlled, dose-response study. Psychoneuroendocrinology.

[7] Zhao J, Song X, Ai X, Gu X, Huang G, Li X (2015). Adjunctive aripiprazole treatment for risperidone-induced hyperprolactinemia: an 8-week randomized, open-label, comparative clinical trial. PLoS One.

[8] Takeuchi H, Remington G (2013). A systematic review of reported cases involving psychotic symptoms worsened by aripiprazole in schizophrenia or schizoaffective disorder. Psychopharmacology.

[9] Takeuchi H, Fathi A, Thiyanavadivel S, Agid O, Remington G (2018). Can aripiprazole worsen psychosis in schizophrenia? A meta-analysis of double-blind, randomized, controlled trials. J Clin Psychiatry.

[10] Chan HY, Lin WW, Lin SK, Hwang TJ, Su TP, Chiang SC (2007). Efficacy and safety of aripiprazole in the acute treatment of schizophrenia in Chinese patients with risperidone as an active control: a randomized trial. J Clin Psychiatry.

[11] Li H, Luo J, Wang C, Xie S, Xu X, Wang X (2014). Efficacy and safety of aripiprazole in Chinese Han schizophrenia subjects: a randomized, double-blind, active parallel-controlled, multicenter clinical trial. Schizophr Res.

[12] Murray RM, Quattrone D, Natesan S, van Os J, Nordentoft M, Howes O (2016). Should psychiatrists be more cautious about the long-term prophylactic use of antipsychotics?. Brit J Psychiat.

[13] Chouinard G, Samaha AN, Chouinard VA, Peretti CS, Kanahara N, Takase M (2017). Antipsychotic-induced dopamine supersensitivity psychosis: pharmacology, criteria, and therapy. Psychother Psychosom.

[14] Fitzgerald P, Dinan TG (2008). Prolactin and dopamine: what is the connection? A review article. J Psychopharmacol (Oxf).

[15] Safer DJ, Calarge CA, Safer AM (2013). Prolactin serum concentrations during aripiprazole treatment in youth. J Child Adolesc Psychopharmacol.

[16] Lozano R, Marin R, Santacruz MJ (2014). Prolactin deficiency by aripiprazole. J Clin Psychopharmacol.

[17] Propst AJ, Jarvis GE, Margolese HC (2016). Aripiprazole-induced hypoprolactinemia in an adult male with first-episode psychosis. Clin Schizophr Relat Psychoses.

[18] Sogawa R, Shimomura Y, Minami C, Maruo J, Kunitake Y, Mizoguchi Y (2016). Aripiprazole-associated hypoprolactinemia in the clinical setting. J Clin Psychopharmacol.

[19] Ohta H, Inoue S, Hara K, Watanabe A (2017). TSH and PRL, side-effect markers in aripiprazole treatment: adjunctive aripiprazole-induced thyrotropin oversuppression in a young man with schizophrenia. BMJ Case Rep.

[20] Faraone SV, Brown WA, Laughren TP (1987). Serum neuroleptic levels, prolactin levels, and relapse: a two-year study of schizophrenic outpatients. J Clin Psychiatry.

[21] Kirkpatrick B, Carpenter WT, Maeda K, Buchanan RW, Breier A, Tamminga CA (1992). Plasma prolactin as a predictor of relapse in drug-free schizophrenic outpatients. Biol Psychiatry.

[22] Appleberg B, Katila H, Rimon R (2000). Inverse correlation between hallucinations and serum prolactin in patients with non-affective psychoses. Schizophr Res.

[23] Segal M, Avital A, Rojas M, Hausvater N, Sandbank S, Liba D (2004). Serum prolactin levels in unmedicated first-episode and recurrent schizophrenia patients: a possible marker for the disease's subtypes. Psychiatry Res.

[24] Segal M, Avital A, Berstein S, Derevenski A, Sandbank S, Weizman A (2007). Prolactin and estradiol serum levels in unmedicated male paranoid schizophrenia patients. Prog Neuro-Psychopharmacol Biol Psychiatry.

[25] Howes OD, Kapur S (2009). The dopamine hypothesis of schizophrenia: version III--the final common pathway. Schizophr Bull.

[26] Rajkumar RP (2014). Prolactin and psychopathology in schizophrenia: a literature review and reappraisal. Schizophr Res Treat.

[27] Hwang T-J, Lo W-M, Chan H-Y, Lin C-F, Hsieh MH, Liu C-C (2015). Fast versus slow strategy of switching patients with schizophrenia to aripiprazole from other antipsychotics. J Clin Psychopharmacol.

[28] Buchanan RW, Kreyenbuhl J, Kelly DL, Noel JM, Boggs DL, Fischer BA (2010). The 2009 schizophrenia PORT psychopharmacological treatment recommendations and summary statements. Schizophr Bull.

[29] Cheng J, Ho H, Chang C, Lan SY (1996). Positive and Negative Syndrome Scale (PANSS): establishment and reliability study of a Mandarin Chinese language version. Chin Psychiatry.

[30] van der Gaag M, Cuijpers A, Hoffman T, Remijsen M, Hijman R, de Haan L (2006). The five-factor model of the positive and negative syndrome scale I: confirmatory factor analysis fails to confirm 25 published five-factor solutions. Schizophr Res.

[31] Wu BJ, Lan TH, Hu TM, Lee SM, Liou JY (2015). Validation of a five-factor model of a Chinese Mandarin version of the Positive and Negative Syndrome Scale (CMV-PANSS) in a sample of 813 schizophrenia patients. Schizophr Res.

[32] Kubo M, Koue T, Maune H, Fukuda T, Azuma J (2007). Pharmacokinetics of aripiprazole, a new antipsychotic, following oral dosing in healthy adult Japanese volunteers: influence of CYP2D6 polymorphism. Drug Metab Pharmacok.

[33] Kishimoto T, Agarwal V, Kishi T, Leucht S, Kane JM, Correll CU (2013). Relapse prevention in schizophrenia: a systematic review and meta-analysis of second-generation antipsychotics versus first-generation antipsychotics. Mol Psychiatry.

[34] Cookson J, Hodgson R, Wildgust HJ (2012). Prolactin, hyperprolactinaemia and antipsychotic treatment: a review and lessons for treatment of early psychosis. J Psychopharmacol.

[35] Lieberman JA, Tollefson G, Tohen M, Green AI, Gur RE, Kahn R (2003). Comparative efficacy and safety of atypical and conventional antipsychotic drugs in first-episode psychosis: a randomized, double-blind trial of olanzapine versus haloperidol. Am J Psychiatry.

[36] Green AI, Lieberman JA, Hamer RM, Glick ID, Gur RE, Kahn RS (2006). Olanzapine and haloperidol in first episode psychosis: two-year data. Schizophr Res.

[37] Ferrier IN, Johnstone EC, Crow TJ, AAPS J (1984). Hormonal effects of apomorphine in schizophrenia. Br J Psychol.

[38] Petty RG (1999). Prolactin and antipsychotic medications: mechanism of action. Schizophr Res.

[39] Kulkarni J, Keks NA, Stuart G, Mackie B, Minas IH, Singh BS (1990). Relationship of psychotic symptoms to haloperidol-stimulated prolactin release. Acta Psychiatr Scand.

[40] Nerozzi D, Magnani A, Sforza V, Scaramucci E, Cerilli M, Moretti C (1990). Plasma prolactin response to domperidone in acute schizophrenia and schizophreniform illness. Psychiatry Res.

[41] Pariante CM (2008). Pituitary volume in psychosis: the first review of the evidence. J Psychopharmacol.

