# Supplementary material for: Abnormally low prolactin levels in schizophrenia patients after switching to aripiprazole in a randomized trial: a biomarker for rebound in psychotic symptoms?
Source: BMC Psychiatry. 2020 Nov 23;20:552. doi: 10.1186/s12888-020-02957-7 (PMC7686669; doi:10.1186/s12888-020-02957-7)
Supplement: Supplementary file 1 — Additional file 1: Table S1. Distribution of the pre-switching antipsychotics and the prolactin level at baseline between the two groups classified by the grouping of prolactin levels at follow up. Table S2. Using separate criterion on sex (< 3.57 ng/mL for men; < 6.12 ng/mL for women) to evaluated the correlation between the abnormal decline in prolactin levels and rebound in psychotic symptoms in patients with schizophrenia. Table S3. Using less stringent criterion of rebound in positive symptoms (PANSS positive subscores increased at least 1 points between adjacent time points) to evaluated the correlation between the abnormal decline in prolactin levels and rebound in psychotic symptoms in patients with schizophrenia. Table S4. Using more stringent criterion of rebound in psychotic positive symptoms (PANSS positive subscores increased at least 3 points between adjacent time points) to evaluated the correlation between the abnormal decline in prolactin levels and rebound in psychotic symptoms in patients with schizophrenia. Table S5. Multivariable logistic regression analysis of correlation between the abnormal decline in prolactin levels and rebound in psychotic positive symptoms (increasing at least 20% between adjacent time points) in patients with schizophrenia (N = 63). Figure S1. Each panel shows each participant’s changes in prolactin serum levels (baseline, 14th Day, and 56th Day) and PANSS positive subscores (baseline, 7th Day, 14th Day, 28th Day, and 56th Day) along the time axis of days in our group’s previous trial. Blue line presents the trend of prolactin serum levels, and red line presents the trend of PANSS positive subscore. [file 12888_2020_2957_MOESM1_ESM.docx]

**Supplementary material**

Supplementary tables and figures

Title:

**Abnormally low prolactin levels in schizophrenia patients after switching to aripiprazole: a biomarker for rebound in psychotic symptoms?**

Ya-Wen Jen^1^, Tzung-Jeng Hwang^2^, Hung-Yu Chan^2,3^, Ming H. Hsieh^2^, Chen-Chung Liu^2^, Chih-Min Liu^2^, Hai-Gwo Hwu^2^, Ching-Hua Kuo^4^, Yi-Ting Lin^2^, Yi-Ling Chien^2^, Wei J. Chen^1,2,5,6*^

^1^ Institute of Epidemiology and Preventive Medicine, College of Public Health, National

Taiwan University, Taipei, Taiwan

^2^ Department of Psychiatry, College of Medicine and National Taiwan University Hospital,

National Taiwan University, Taipei, Taiwan

^3^ Office of Superintendent, Taoyuan Psychiatric Center, Ministry of Health and Welfare, Taoyuan City, Taiwan

^4^ School of Pharmacy, College of Medicine, National Taiwan University, Taipei, Taiwan

^5^ Centers for Genomic and Precision Medicine, National Taiwan University, Taipei, Taiwan

^6^ Center for Neuropsychiatric Research, National Health Research Institutes, Miaoli, Taiwan

* Corresponding author: Wei J. Chen, 17 Xu-Zhou Road, Taipei 100, Taiwan ([wjchen@ntu.edu.tw](mailto:wjchen@ntu.edu.tw)).

**Table S1**. Distribution of the pre-switching antipsychotics and the prolactin level at baseline between the two groups classified by the grouping of prolactin levels at follow up.

**Table S2.** Using separate criterion on sex (< 3.57 ng/mL for men; < 6.12 ng/mL for women) to evaluated the correlation between the abnormal decline in prolactin levels and rebound in psychotic symptoms in patients with schizophrenia.

**Table S3.** Using less stringent criterion of rebound in positive symptoms (PANSS positive subscores increased at least 1 points between adjacent time points) to evaluated the correlation between the abnormal decline in prolactin levels and rebound in psychotic symptoms in patients with schizophrenia.

**Table S4.** Using more stringent criterion of rebound in psychotic positive symptoms (PANSS positive subscores increased at least 3 points between adjacent time points) to evaluated the correlation between the abnormal decline in prolactin levels and rebound in psychotic symptoms in patients with schizophrenia.

**Table S5.** Multivariable logistic regression analysis of correlation between the abnormal decline in prolactin levels and rebound in psychotic positive symptoms (increasing at least 20% between adjacent time points) in patients with schizophrenia (N = 63).

**Figure S1.** Each panel shows each participant’s changes in prolactin serum levels (baseline, 14th Day, and 56th Day) and PANSS positive subscores (baseline, 7th Day, 14th Day, 28th Day, and 56th Day) along the time axis of days in our group’s previous trial. Blue line presents the trend of prolactin serum levels, and red line presents the trend of PANSS positive subscore.

| **Table S1.** Distribution of the pre-switching antipsychotics and the prolactin level at baseline between the two groups classified by the grouping of prolactin levels at follow up. | | | | | | | | | | | | | | |  | |  |
| --- | --- | --- | --- | --- | --- | --- | --- | --- | --- | --- | --- | --- | --- | --- | --- | --- | --- |
| Pre-switching antipsychotics use | Abnormally low prolactin level at follow up  (N = 25) | | | |  | Normal prolactin levels at follow-up  (N = 38) | | | |  | Total  (N = 63) | | | |  | |  |
|  | Prolactin level at baseline, ng/dL, mean (SD) | | n (%) | |  | Prolactin level at baseline, ng/dL, mean (SD) | | n (%) | | | Prolactin level at baseline, ng/dL, mean (SD) | | n (%) | |  | |  |
| First generation antipsychotics | 17.5 | (14.7) | 11 | (44) |  | 87.1 | (74.8) | 14 | (37) | |  |  | 25 | (40) | |  |  |
| Second generation antipsychotics |  |  |  |  |  |  |  |  |  | |  |  |  |  | |  |  |
| Risperidone | 15.5 | (9.1) | 2 | (8) |  | 73.9 | (65.9) | 7 | (18) | | 60.9 | (62.7) | 9 | (14) | |  |  |
| Amisulpride | 62.8 | (48.3) | 2 | (8) |  | 129.7 | (79.7) | 5 | (13) | | 110.6 | (75.5) | 7 | (11) | |  |  |
| Olanzapine | 18.3 | (11.9) | 7 | (28) |  | 32.3 | (42.7) | 5 | (13) | | 24.2 | (28.1) | 12 | (19) | |  |  |
| Zotepine | 35.1 | - | 1 | (4) |  | 64.5 | (66.6) | 3 | (8) | | 57.1 | (56.3) | 4 | (6) | |  |  |
| Ziprasidone | 3.2 | - | 1 | (4) |  | 47.1 | - | 1 | (3) | | 25.1 | (31.1) | 2 | (3) | |  |  |
| Quetiapine | 4.7 | - | 1 | (4) |  | 8.1 | (2.5) | 3 | (8) | | 7.2 | (2.7) | 4 | (6) | | | |

| **Table S2.** Multivariable logistic regression analysis of rebound in positive subscores on the abnormally low prolactin levels (< 3.57 ng/mLfor men; < 6.12 ng/mL for women)  among schizophrenia patients participating in the trial of switching to aripiprazole (N = 63) | | | | | | | | | | |  |
| --- | --- | --- | --- | --- | --- | --- | --- | --- | --- | --- | --- |
| Variables | Rebound in  positive subscores (n = 21) | | No rebound in positive subscores (n = 42) | | |  | | Multivariate-adjusted  OR (95% CI) | | |  |
|  |  |  |  |  |  |  |  | Model 1 | | Model 2 |  |
| Male, n (%), (ref. female) | 9 | (42.90) | | 17 | (40.50) | | 0.89 (0.29-2.80) | | 0.65 (0.16-2.66) | |  |
| Early age of onset, n (%), (ref. late age of onset)^a^ | 2 | (9.52) | | 6 | (14.29) | | 0.54 (0.09-3.17) | | 0.35 (0.06-2.25) | |  |
| Preswitching medication |  |  | |  |  | |  | |  | |  |
| First generation antipsychotics, n (%) | 12 | (57.10) | | 13 | (31.00) | | 1.00 (reference) | | 1.00 (reference) | |  |
| Second generation antipsychotics, n (%) | 9 | (42.90) | | 29 | (69.00) | | 0.33 (0.12-0.99) | | 0.23 (0.07-0.82) | |  |
| Prolactin level at baseline, ng/dL, mean (SD) | 47.6 | (69.48) | | 55.6 | (57.63) | | - | | 1.00 (0.98-1.01) | |  |
| Positive subscore in PANSS at baseline, mean (SD)b | 12.2 | (4.41) | | 9.4 | (4.26) | | - | | 1.23 (1.06-1.42) | |  |
|  |  |  | |  |  | |  | |  | |  |
| Abnormally low prolactin levels at follow up, n (%) | 12 | (57.10) | | 13 | (31.00) | | 1.35 (0.44-4.12) | | 1.54 (0.41-5.75) | |  |
| Interaction term, abnormally low prolactin levels × sex |  |  | |  |  | | 1.02 (0.57-1.81) | | 1.13 (0.59-2.17) | |  |
| Model 1: adjustment for sex, early age of onset, and pre-switching medication  Model 2: Model 1 plus adjustment for prolactin level and positive subscore in PANSS at baseline  ^a^≤ 18 years old defined as early age of onset; One observation was deleted due to missing value  ^b^Including delusions (p1), hallucinations (p3), grandiosity (p5), suspiciousness (p6),and unusual thought content (g9) *p < .05 | | | | | | | | | | | |

| **Table S3**. Multivariable logistic regression analysis of correlation between the abnormally low in prolactin levels and rebound in psychotic positive symptoms (increasing at least 1 points between adjacent time points) in patients with schizophrenia (N = 63) | | | | | | | | |  |
| --- | --- | --- | --- | --- | --- | --- | --- | --- | --- |
| Variable | Rebound in PANSS  positive subscores (n = 33) | | | No rebound in PANSS positive subscores (n = 30) | | Adjusted OR^a^ | 95% CI | |  |
| Male, n (%), (ref. female) | 15 | (57.69) | 18 | | (48.65) | 0.76 | | 0.23-2.53 | |
| Early age of onset, n (%), (ref. late age of onset)^b,c^ | 4 | (12.12) | 4 | | (13.33) | 0.99 | | 0.20-5.02 | |
| Preswitching medication |  |  |  | |  |  | |  | |
| First generation antipsychotics, n (%) | 17 | (51.52) | 8 | | (26.67) | 1.00 | | - | |
| Second generation antipsychotics, n (%) | 16 | (48.48) | 22 | | (73.33) | 0.33 | | 0.11-1.02 | |
|  |  |  |  | |  |  | |  | |
| Abnormal decline in prolactin levels, n (%), (ref. normal decline) | 18 | (54.55) | 7 | | (23.33) | 4.42* | | 1.29-15.2 | |
| ^a^Adjusted for sex, age of onset, and pre-switching medication  ^b^≤ 18 years old defined as early age of onset ^c^One observation was deleted due to missing value  *p < .05 | | | | | | | | |  |

| **Table S4.** Multivariable logistic regression analysis of correlation between the abnormally low in prolactin levels and rebound in psychotic positive symptoms (increasing at least 3 points between adjacent time points) in patients with schizophrenia (N = 63) | | | | | | | | |
| --- | --- | --- | --- | --- | --- | --- | --- | --- |
| Variable | Rebound in PANSS  positive subscores (n = 14) | | No rebound in PANSS positive subscores  (n = 49) | | | Adjusted OR^a^ | 95% CI |  |
| Male, n (%), (ref. female) | 6 | (42.86) | | 20 | (40.82) | 0.57 | 0.13-2.42 |  |
| Early age of onset, n (%), (ref. late age of onset)^b,c^ | 1 | (7.14) | | 7 | (14.29) | 0.40 | 0.04-4.14 |  |
| Preswitching medication |  |  | |  |  |  |  |  |
| First generation antipsychotics, n (%) | 9 | (64.29) | | 16 | (32.65) | 1.00 | - |  |
| Second generation antipsychotics, n (%) | 5 | (35.71) | | 33 | (67.35) | 0.24 | 0.07-0.90 |  |
|  |  |  | |  |  |  |  |  |
| Abnormal decline in prolactin levels, n (%), (ref. normal decline) | 8 | (57.14) | | 17 | (34.69) | 2.89 | 0.71-11.8 |  |
| ^a^Adjusted for sex, age of onset, and pre-switching medication  ^b^≤ 18 years old defined as early age of onset ^c^One observation was deleted due to missing value  *p < .05 | | | | | | | |  |

| **Table S5.** Multivariable logistic regression analysis of correlation between the abnormal decline in prolactin levels and rebound in psychotic positive symptoms (increasing at least 20% between adjacent time points) in patients with schizophrenia (N = 63) | | | | | | | | | |
| --- | --- | --- | --- | --- | --- | --- | --- | --- | --- |
| Variable | Rebound in PANSS  positive subscores (n = 21) | | No rebound in PANSS positive subscores  (n = 42) | | | Adjusted OR^a^ | 95% CI | |  |
| Male, n (%), (ref. female) | 7 | (33.33) | | 19 | (45.24) | 0.22 | 0.05-0.96 |  |  |
| Early age of onset, n (%), (ref. late age of onset)^b,c^ | 1 | (4.76) | | 7 | (16.67) | 0.19 | 0.02-2.17 |  |  |
| Preswitching medication |  |  | |  |  |  |  |  |  |
| First generation antipsychotics, n (%) | 11 | (52.38) | | 14 | (33.33) | 1.00 | - |  |  |
| Second generation antipsychotics, n (%) | 10 | (47.62) | | 28 | (66.67) | 0.34 | 0.10-1.14 |  |  |
|  |  |  | |  |  |  |  |  |  |
| Abnormal decline in prolactin levels, n (%), (ref. normal decline) | 12 | (57.14) | | 13 | (30.95) | 5.27 | 1.33-20.9 |  |  |
| ^a^Adjusted for sex, age of onset, and pre-switching medication  ^b^≤ 18 years old defined as early age of onset ^c^One observation was deleted due to missing value  *p < .05 | | | | | | | |  |  |


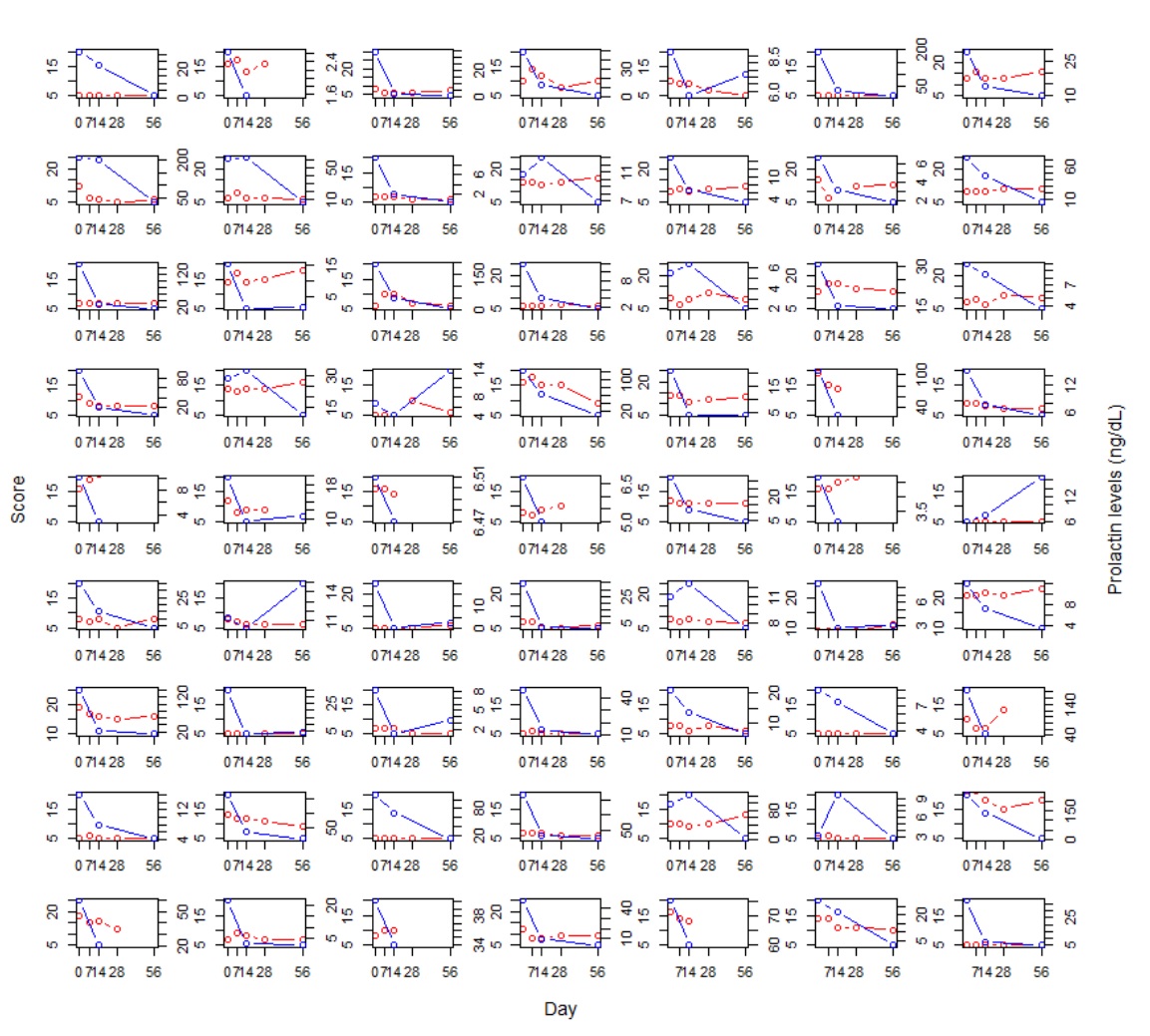
**Figure S1**. Each panel shows each participant’s changes in prolactin serum levels (baseline, 14^th^ Day, and 56^th^ Day) and PANSS positive subscores (baseline, 7^th^ Day, 14^th^ Day, 28^th^ Day, and 56^th^ Day) along the time axis of days in our group’s previous trial. Blue line presents the trend of prolactin serum levels, and red line presents the trend of PANSS positive subscore.
